# HIV Treatment as Prevention: Debate and Commentary—Will Early Infection Compromise Treatment-as-Prevention Strategies?

**DOI:** 10.1371/journal.pmed.1001232

**Published:** 2012-07-10

**Authors:** Myron S. Cohen, Christopher Dye, Christophe Fraser, William C. Miller, Kimberly A. Powers, Brian G. Williams

**Affiliations:** 1 Department of Microbiology and Immunology, University of North Carolina, Chapel Hill, North Carolina, United States of America; 2 Department of Medicine, University of North Carolina, Chapel Hill, North Carolina, United States of America; 3 Department of Epidemiology, University of North Carolina, Chapel Hill, North Carolina, United States of America; 4 World Health Organization, Geneva, Switzerland; 5 Medical Research Council Centre for Outbreak Analysis and Modelling, Department of Infectious Disease Epidemiology, Imperial College London, London, United Kingdom; 6 South African Centre for Epidemiological Modelling and Analysis, Stellenbosch, South Africa; Duke University Medical Center, United States of America

## Abstract

Universal HIV testing and immediate antiretroviral therapy for infected individuals has been proposed as a way of reducing the transmission of HIV and thereby bringing the HIV epidemic under control. It is unclear whether transmission during early HIV infection—before individuals are likely to have been diagnosed with HIV and started on antiretroviral therapy—will compromise the effectiveness of treatment as prevention. This article presents two opposing viewpoints by Powers, Miller, and Cohen, and Williams and Dye, followed by a commentary by Fraser.

## Introduction to the Debate

Triple-combination antiretroviral therapy (ART) first became available in 1995 for the treatment of people living with HIV [1]. The fact that ART reduces viral load raised the prospect of using ART not only to keep people alive, but also to control the epidemic by annually testing everyone at risk of HIV infection and immediately starting infected individuals on ART [2]–[4]. In 2011, the HPTN 052 trial showed that ART reduced the risk of infection in heterosexual HIV-serodiscordant couples (where one individual is HIV-seropositive and the other is not) by 96% (95% confidence interval [CI], 73% to 99%) and decisively confirmed the impact of treatment on heterosexual transmission [5].

If the individual-level effects observed in the HPTN 052 trial are to be successfully replicated at a population level, many operational issues need to be addressed. An issue of particular importance concerns transmission in the early stages of HIV infection, before an individual is likely to be diagnosed with HIV and start receiving treatment. Early HIV infection (EHI), the first 3–6 months after infection, includes acute HIV infection (AHI), the period before the development of antibodies to HIV, when the concentration of virus in the plasma spikes and then falls to the set-point viral load. Chronic HIV infection (CHI) comprises an asymptomatic period following EHI, characterized by a stable viral load (∼10^4.5^ copies/ml) and relatively low transmissibility, as well as late infection/AIDS, when viral load and transmissibility are elevated again. Different studies have arrived at widely differing estimates of the proportion of HIV transmission events that occur during the first 3–6 months after HIV infection, ranging from 5% to 95% [6]. High levels of HIV transmission early in infection could compromise the impact of universal testing and treatment on HIV transmission at a population level, so it is essential to resolve this issue if ART is to be used to help control the HIV pandemic.

In this debate, which specifically addresses heterosexual transmission of HIV, Powers, Miller, and Cohen argue that up to 40% of transmission takes place during EHI, and that this transmission will compromise the effectiveness of treatment as prevention. Williams and Dye argue that only about 2% of transmission takes place during AHI, so that annual testing and immediate ART will be sufficient to control the epidemic of HIV. In the final section, Christophe Fraser summarizes and weighs in on the debate.

## Kimberly A. Powers, William C. Miller, Myron S. Cohen's Viewpoint: Acute and Early HIV Infection Will Limit the Effectiveness of HIV Treatment as Prevention

Although the use of ART to stop the spread of HIV has become a major focus of HIV prevention, reliable empirical evidence to support this strategy at the population level does not exist, and its success in the real world may be limited by numerous factors [7]. Here we focus on one particular factor: transmission during AHI and EHI, which will not be affected by a treatment-as-prevention approach. We argue that high levels of transmission during this period of maximal infectiousness [6] will compromise the effectiveness of HIV treatment as prevention.

### Transmission Biology

HIV acquisition leads to a ramp-up in viremia to 10 million copies/ml or more [8], with a subsequent cell-mediated immune response that leads to decreased viral replication during asymptomatic infection [9]. The best available estimates of heterosexual HIV transmission by infection stage, calculated among steady couples in Rakai, Uganda, suggest that transmissibility is 26 times as high (95% CI, 13–54) during EHI as it is in the subsequent asymptomatic period [10]. Among the Rakai couples, the probability of a newly infected person transmitting HIV to his or her partner in the first five months of infection was estimated at 43% [11].

Importantly, the elevation in transmissibility observed during EHI in Rakai is greater than would be expected based on viral load alone [10]. If viral load were the only driver of infectiousness, then we would expect transmission rates during AHI and EHI to be only a few times higher than during chronic infection, as Williams and Dye describe below. The mechanism for the additional enhancement in transmissibility observed during EHI in Rakai has not been elucidated, but there is evidence from macaques that individual virions from EHI are 75–750 times as infectious as virions from CHI [12].

### Mathematical Modelling

Mathematical modelers have attempted to predict the potential population benefits of ART [13]. With perhaps the most optimistic model, Granich et al. have argued that universal annual HIV testing and immediate ART would lead to HIV “elimination,” defined as one incident infection per 1,000 persons annually, within ten years in South Africa [4]. However, the analyses leading to this conclusion failed to account for the effect of poor engagement in care [14] and the increased infectiousness of persons with EHI [15], who would not be reached by the test-and-treat strategy.

Modelling estimates of the percentage of new cases that are due to contact with EHI index cases vary widely, depending on epidemic stage, model structure, transmission mode, and EHI definition. Most endemic-phase estimates have been in the range of 5% to 40% [6], broadly consistent with estimates of 25%–50% from phylogenetic studies [16]. However, the data available for parameterizing most of these models have been limited. Using behavioral and viral load data from Lilongwe, Malawi, as well as the best available estimates of transmission efficiency by infection stage [10], we constructed a mathematical model that allowed for transmission both within and outside of steady heterosexual relationships, substantial variation in transmissibility over the course of infection, and heterogeneity in behavioral risk (rates of partner change and contact within pairs) [15]. We used a Bayesian melding procedure to account for input uncertainty, to fit the model to empirical HIV prevalence data from Lilongwe, and to express uncertainty around outputs.

In our model, we estimated that transmission rates were elevated for 4.8 months (i.e., EHI lasted for 4.8 months) and that 38% (95% credible interval 19%–52%) of endemic-phase incident infections arose from contact with EHI index cases annually. We estimated that an annual test-and-treat strategy with very optimistic chronic-phase coverage and engagement levels of 90% or greater could substantially reduce HIV incidence, and eventually prevalence, in this setting, but HIV elimination was possible only at coverage and engagement levels of 99% or greater. This prediction is consistent with the conclusion of Granich et al. [4] and Williams and Dye (below) that with truly universal coverage and engagement, annual test-and-treat strategies could lead to HIV elimination. At more realistic coverage and engagement levels (75%–85% of chronic-phase cases), however, elimination did not occur in our model, but additional interventions halting transmission during EHI led to marked, durable reductions in HIV prevalence and incidence. Even in sensitivity analyses where the contribution of EHI in Lilongwe was only half our best estimate of 38% (19%), our results suggested that unless test-and-treat coverage is essentially perfect, the impact of such interventions is likely to be limited substantially by transmission during EHI.

Williams et al. [17] recently argued (as Williams and Dye argue below) that the 38% of cases we estimated to arise from contact with EHI index cases is too high, proposing instead that only 2%–4% of incident infections arise during AHI. By basing their calculations only on the putative relationship between chronic-phase viral load and transmissibility, they do not capture the greater-than-expected transmissibility observed during EHI among the Rakai couples [10]. Furthermore, the duration (one month) and increase in transmission rate per sexual encounter (three-fold) that they calculate for AHI correspond to an expected within-couple transmission probability of ∼3% during AHI (calculated as 1−e^−βτ*d*^, where β = 0.106 cases per person-year, the asymptomatic-period transmission rate estimated from the Rakai data [10]; τ = 3, the proposed relative increase in transmissibility comparing AHI and asymptomatic infection [17] and below; and *d* = 1 month, the proposed duration of AHI [17] and below). This within-couple transmission probability of 3% during AHI is dramatically lower than the 43% observed during EHI in Rakai [11]. Simply put, the calculations of Williams and Dye are inconsistent with the best available data from epidemiological, mathematical, and phylogenetic studies regarding transmission during EHI [6],[10],[11],[16].

### Implications for Treatment as Prevention

We believe that EHI can be expected to limit the impact of treatment-as-prevention programs—at least in settings similar to Lilongwe, Malawi—and that reductions in HIV incidence and prevalence can be optimized through intervention packages that stop transmission during both CHI and EHI. A number of randomized trials to investigate the population-level effects of treatment as prevention are underway [18]. Because they do not include a specific strategy for dealing with AHI or EHI, the extent to which they succeed will provide some indication as to whether or not transmission during EHI compromises the effectiveness of treatment as prevention. In addition, some of these studies will use phylogenetic measurements to clarify transmission events attributable to acute/early cases versus chronic cases, providing more specific information about the importance of AHI/EHI in the context of these trials. If AHI and EHI are found to limit treatment as prevention empirically, we will need to develop a more efficient strategy for identifying individuals with EHI, as well as credible behavioral and/or treatment-based intervention strategies [19] for this period.

## Brian G. Williams and Christopher Dye's Viewpoint: Acute and Early HIV Infection Will Not Limit the Effectiveness of HIV Treatment as Prevention

It has been shown that successful ART reduces heterosexual transmission of HIV by 96% (95% CI, 73%–99%) [5], more than enough to eliminate HIV transmission [4]. However, Powers et al. ([15] and above) argue that 38% of transmission events occur during the first 4.8 months after HIV acquisition and that unless HIV-positive individuals start ART very early in this period, ART will not be sufficient to eliminate HIV transmission. Here we argue that (1) there is no convincing evidence to support their estimate of the proportion of transmission events that take place during the first 4.8 months, and (2) even if their estimate is correct, annual testing and immediate treatment would nevertheless be sufficient to eliminate transmission

### Transmission during Early HIV Infection

Twelve studies, summarized by Cohen et al. [6], suggest that between 8% and 75% of new infections occur during EHI. Unfortunately, all of the studies that are concerned with heterosexual transmission depend on one set of data collected from the retrospective identification of 23 couples in Rakai, Uganda [11], in a study designed for other purposes. In ten couples in the Rakai study, both partners seroconverted in the same ten-month interval between testing. It was assumed that the first person in the couple was infected after an average of five months, leaving five months for them to infect their partner. Of the 13 remaining serodiscordant couples, three of the seronegative partners were infected in the next ten months, giving a rate ratio for infection during the first and second periods of 7.3 (95% CI, 3.1–17.3) [11]. Allowance was made for the self-reported number of sexual encounters, but not for the possibility that the second person was infected from outside the relationship. Data from a study of 23 couples, designed for other purposes, in which people were tested for HIV only at ten-month intervals and were identified retrospectively, which relied on self-reported sexual activity, and which did not determine whether or not the infection came from outside the relationship [11], do not provide a sound basis for drawing conclusions about the importance of EHI.

Since there is no convincing direct evidence that heterosexual transmission is higher during AHI than during the asymptomatic period of CHI, we consider indirect estimates based on viral load and the likely duration of AHI. Most new HIV infections are established by a single founder virus. The concentration of virions in the plasma then increases rapidly over three to four weeks, reaching ∼10^6.5^ copies/ml, and then falls equally rapidly to a set point at ∼10^4.5^ copies/ml [20],[21]. From a preliminary analysis of data presented by Robb [20] for people in the acute phase of infection, the peak concentration of virus in the plasma is 10^2.1^ (95% CI,10^1.6^–10^2.5^) copies/ml times greater than at the set point, and AHI lasts for 2.1 (1.6–2.5) weeks. Miller et al. [16] likewise observe that “acute HIV infection, when the concentration of HIV in blood and genital secretions is extremely high, is only a few weeks in duration.” According to the model of Powers et al. (Supplementary Web Appendix Figure 1 in [15]), AHI , the period of peak viral load that lasts for a maximum of six weeks, corresponding to an average duration, with the same area under the curve, of two weeks, with average viral load increased about 20-fold.

Transmission increases with viral load, and most authors assume that transmission increases as viral load to the power of 0.3 to 0.5 [22]–[24]; the relationship is clearly sublinear so that transmission saturates as viral load increases [25]. A more biologically plausible model [26], which gives an equally good fit to the available data [27], assumes that transmission increases linearly with viral load at low values of viral load, but converges to an asymptote above a viral load of 10^4.4^ copies/ml [27]. In order to estimate *P_i_*, the proportion of infections that take place in stage *i*, we calculate, to first order,
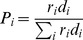
(1)where *d_i_* is the duration and *r_i_* is the relative infectiousness of stage *i*, assuming a steady state, in which prevalence, incidence, and mortality are constant, and random mixing. With a mean set-point viral load of 10^4.5^ copies/ml, an increase in the average viremia from 10^4.5^ copies/ml to 10^6.5^ copies/ml during AHI would make little difference to the overall rate of transmission. Even if we generously assume that the viral load peak during AHI lasts for one month and that transmission rates per sexual encounter are increased three-fold during AHI, Equation 1 shows that AHI accounts for only 2% of all transmissions and would be consequential only if people had several partners in the two-week period of AHI, which is not supported by data. Raised viremia during AHI does not support the claim that EHI contributes significantly to heterosexual HIV transmission.

Powers et al. [15] estimate that EHI lasts for 4.8 months and that during this time the risk of infection per sexual encounter is 30.3 (13.6–47.1) times greater than it is during the asymptomatic period of CHI. If we grant these assumptions, Equation 1, which assumes a steady state, shows that about 56% of infections would then occur during EHI, in agreement with their estimate of 78% (95% credible interval, 68%–85%) in 1975, falling to 38% (19%–52%) in 2010. The agreement between this estimate using Equation 1 and the estimate of Powers et al. [15] shows that our different conclusions arise from our different estimates of the duration of elevated infectiousness and transmission rates during that period, and is not due to other structural details of the model.

### Early Treatment and *R*_0_

Even if the modeled outcomes of Powers et al. [15] are correct, annual testing and immediate treatment would still be sufficient to eliminate transmission. The initial doubling time of the prevalence of HIV in the Malawi study was 1.3 years ([15]), and the greater the relative risk of transmission in EHI, the smaller must be the value of the basic reproduction number, *R*_0_, to maintain the same initial doubling time, as follows directly from the Euler-Lotka equation [28],[29]. If we suppose that transmission per sexual contact is 30 times higher during EHI than during the next ten years of CHI, as proposed by Powers et al. [15], the value of *R*_0_, subject to the constraint that the initial doubling time is 1.3 years, would have to be ∼2 rather than ∼5–10 [27]. Testing people at regular intervals of one year and starting them immediately on ART would reduce *R*_0_ to 0.8 [27]; testing people more frequently would reduce it further. Thus, early treatment could still lead to elimination of HIV transmission, and adding other interventions, such as male circumcision, would increase the impact further.

**Figure 1:**
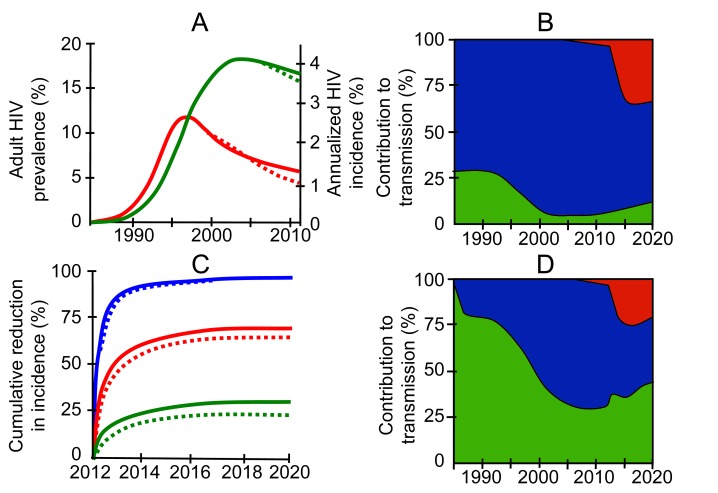
The predicted effect of different levels of acute infection on a combination prevention package including universal testing and treatment, as will be tested in the PopART trial [20]. (A) Green line: prevalence; red line: incidence. Two versions of a model are fitted to the adult HIV prevalence curve for South Africa (Joint United Nations Programme on HIV/AIDS): one “corrected” for serial monogamy effects in low-risk individuals [5], and thus with a low contribution of AHI (solid line), and one without the correction, and thus with a high contribution of AHI (dashed line). Fitted parameters are as follows: the proportion of individuals in three risk groups (low, medium, and high), rate of partner change for high-risk individuals, assortativity of mixing by risk, start time, early treatment rates, and an overall infectiousness parameter. Other parameters were fixed from the literature [13],[14]. (C) The intervention is introduced in 2012, and predictions are made until 2020, for three scenarios ranging from the very pessimistic (green line), through “just on target” (red line), to very optimistic (blue line). The results are surprisingly independent of the amount of transmission from AHI, as indicated by the solid versus dashed lines. (B and D) The contribution to transmission from individuals in different disease stages in the just-on-target scenarios is plotted in (B), corresponding to solid lines in (A) and (C) (corrected for serial monogamy effects), and (D), corresponding to dashed lines in (A) and (C) (not corrected for serial monogamy effects). Shown are all new infections of index cases in AHI and EHI (green), of index cases in untreated CHI (blue), and of index cases in treated CHI (red), as a proportion of total new infections.

### Implications for Treatment as Prevention

There is agreement that ART reduces the rate of transmission by about 25 times [5] and that this reduction is much greater than has been demonstrated with any other currently available intervention. It is unlikely that AHI or EHI significantly compromises the impact of treatment on transmission. We agree that if the intention is to start people on ART as soon as possible after they become infected with HIV, ways of detecting people in the acute phase of HIV infection would increase the impact of treatment as prevention. Whatever may be the precise details of transmission during AHI, treatment as prevention must now be the cornerstone of HIV prevention programs. Going beyond mathematical modelling, the magnitude of the effects of treatment as prevention are being evaluated in a number of field trials [18]. We expect the results of these trials to offer, for the first time, the prospect of an AIDS-free generation [30],[31].

## Christophe Fraser's Commentary on the Debate

The role of AHI and EHI in transmission has been debated since the early days of the HIV epidemic [32] and for much the same reason is still debated today: it seems self-evidently important but is hard to pin down. It is the subject of renewed attention in light of growing interest in treatment as prevention, because unless diagnosis can be made during AHI, most individuals will have passed through EHI before universal testing and treatment would start them on ART.

Powers et al. argue that EHI is a major driver of the epidemic, while Williams and Dye suggest a minimal role for EHI; other studies provide estimates across this range [6]. The debating parties agree that data from the Rakai study in Uganda indicate very high onward transmission in EHI, with 43% of couples found to be mutually infected at the first follow-up after neither of them was [11], and they agree that this is not consistent with expectations from viral load alone [10]. Powers et al. support the epidemiological observation (high transmission) and argue that there is no reason to believe that viral load is a good marker for infectiousness in EHI (true), while Williams and Dye support arguments based on viral load and argue that, with only 23 couples, the Rakai study [11] is, in this context, too small to draw such a strong conclusion (also true).

A pivotal point that neither party delves deeply enough into is the effect of patterns of risk behavior. In a reanalysis of the Rakai data, Hollingsworth et al. [10] show that low-risk (serial monogamy) and high-risk (random mixing) contexts led to significantly different estimates of the extent of transmission during EHI; Eaton et al. [33] show that transmission in a sexual network with concurrent partnerships produces intermediate estimates. Powers et al. obtained higher estimates by allowing for complex correlations between partner change rates and transmission probabilities per sex act [15]. Finally, Koopman et al. [34] emphasize that assuming constant sexual risk behavior over individuals' lifetimes is neither sensible nor supported by the data, and this too plays into the estimation of the role of AHI, since if partner change rates decline with age, EHI becomes more important.

While the role of different patterns of risk behavior in driving EHI may have been underestimated, the argument made by Williams and Dye that AHI and EHI do not matter to prevention efforts as much as we might think may in fact be more fundamental. This argument is based on the Euler-Lotka equation, which constrains the relationship between growth rates and generation times [35],[36]. Here, I test this argument using a conventional mathematical model of HIV transmission, which extends earlier models [4],[37] and is more complex than the models that Williams and Dye have used in this context. Estimates of transmission rates during EHI in the model are based on the data from Rakai, which is still the best evidence to date on this topic, and the model is fit to national surveillance data from South Africa (from the Joint United Nations Programme on HIV/AIDS). The model allows the rate of transmission during EHI to be modified by turning on or off the correction factor for finite partnerships amongst low- and medium-risk individuals [10]; this is more efficient than increasing the parameter for the infectiousness of EHI, due to the counteracting effects of limited partnership turnover on biological infectiousness. When the contribution of EHI is tuned up or down, very different model projections result, as expected.

However, changing our assumptions about the importance of AHI and EHI not only affects our predictions about the future, but also changes our interpretation of what has happened in the past: each time the contribution of EHI is tuned up or down, the model must be refitted to data. Figure 1 shows the outcome of this process: it broadly confirms the prediction of the Euler-Lotka equation in the context of a more complex mathematical model, validating the hypothesis of Williams and Dye that the total effectiveness of treatment as prevention depends surprisingly little on the effect of EHI on transmission.

It must be stated that these predictions are based on a model that is still relatively simple, and reality may yet surprise us. Further modelling work could play a useful role by determining more systematically under which circumstances the prediction of the Euler-Lotka equation is or is not expected to hold, and guiding the collection of appropriate data. Treatment as prevention holds extraordinary promise, but will also be expensive and challenging to deliver in many settings. Arguments about potential barriers to success, such as presented in this debate, need careful consideration. Population-based trials, such as PopART (HPTN 071) [38] and others [18] that are being planned, as well as more observational data, will provide much needed empirical tests of the proposal that treatment as prevention is feasible and effective.

**Key Points**

- Two opposing model-based viewpoints are presented about whether transmission during early HIV infection is likely to compromise the effectiveness of treatment as prevention, i.e., using universal HIV testing and immediate ART to halt the transmission of HIV in a population.
- Powers, Miller, and Cohen's model suggests that 38% of transmission takes place in the first few months after HIV infection, i.e., before infections would be detected and treated via annual testing and treatment, making early HIV transmission a serious impediment to treatment as prevention.
- Williams and Dye argue that their model shows that the high levels of viremia during the acute phase of HIV infection do not significantly increase HIV transmission and that the risk of infection is not significantly higher during early infection than it is during chronic infection. They argue further that even if there were much higher rates of transmission in the acute and early stages of infection, early treatment would still be effective in controlling the epidemic of HIV.
- Fraser highlights that the epidemiological contribution of acute infection depends not just on infectiousness but also on patterns of risk behavior. However, Fraser largely concurs with Williams and Dye that the effect of acute and early infection on the predicted impact of universal testing and treatment may be much smaller than expected.
- All authors agree that future modelling and empirical studies will be useful in elucidating the impact of treatment as prevention on the epidemic of HIV.

## Abbreviations

AHI: acute HIV infection
ART: antiretroviral therapy
CHI: chronic HIV infection
CI: confidence interval
EHI: early HIV infection

## References

[1] Ho DD (1995). Time to hit HIV, early and hard.. N Engl J Med.

[2] Hosseinipour M, Cohen MS, Vernazza PL, Kashuba AD (2002). Can antiretroviral therapy be used to prevent sexual transmission of human immunodeficiency virus type 1?. Clin Infect Dis.

[3] Montaner JS, Hogg R, Wood E, Kerr T, Tyndall M (2006). The case for expanding access to highly active antiretroviral therapy to curb the growth of the HIV epidemic.. Lancet.

[4] Granich RM, Gilks CF, Dye C, De Cock KM, Williams BG (2009). Universal voluntary HIV testing with immediate antiretroviral therapy as a strategy for elimination of HIV transmission: a mathematical model.. Lancet.

[5] Cohen MS, Chen YQ, McCauley M, Gamble T, Hosseinipour MC (2011). Prevention of HIV-1 infection with early antiretroviral therapy.. N Engl J Med.

[6] Cohen MS, Shaw GM, McMichael AJ, Haynes BF (2011). Acute HIV-1 infection.. N Engl J Med.

[7] Smith K, Powers KA, Kashuba AD, Cohen MS (2011). HIV-1 treatment as prevention: the good, the bad, and the challenges.. Curr Opin HIV AIDS.

[8] Piatak M, Saag MS, Yang LC, Clark SJ, Kappes JC (1993). High levels of HIV-1 in plasma during all stages of infection determined by competitive PCR.. Science.

[9] Koup RA, Safrit JT, Cao Y, Andrews CA, McLeod G (1994). Temporal association of cellular immune responses with the initial control of viremia in primary human immunodeficiency virus type 1 syndrome.. J Virol.

[10] Hollingsworth TD, Anderson RM, Fraser C (2008). HIV-1 transmission, by stage of infection.. J Infect Dis.

[11] Wawer MJ, Gray RH, Sewankambo NK, Serwadda D, Li X (2005). Rates of HIV-1 transmission per coital act, by stage of HIV-1 infection, in Rakai, Uganda.. J Infect Dis.

[12] Ma ZM, Stone M, Piatak M, Schweighardt B, Haigwood NL (2009). High specific infectivity of plasma virus from the pre-ramp-up and ramp-up stages of acute simian immunodeficiency virus infection.. J Virol.

[13] Cohen MS, Gay CL (2010). Treatment to prevent transmission of HIV-1.. Clin Infect Dis.

[14] Gardner EM, McLees MP, Steiner JF, Del Rio C, Burman WJ (2011). The spectrum of engagement in HIV care and its relevance to test-and-treat strategies for prevention of HIV infection.. Clin Infect Dis.

[15] Powers KA, Ghani AC, Miller WC, Hoffman IF, Pettifor AE (2011). The role of acute and early HIV infection in the spread of HIV and implications for transmission prevention strategies in Lilongwe, Malawi: a modelling study.. Lancet.

[16] Miller WC, Rosenberg NE, Rutstein SE, Powers KA (2010). Role of acute and early HIV infection in the sexual transmission of HIV.. Curr Opin HIV AIDS.

[17] Williams BG, Granich R, Dye C (2011). Role of acute infection in HIV transmission.. Lancet.

[18] Granich R, Gupta S, Suthar AB, Smyth C, Hoos D (2011). Antiretroviral therapy in prevention of HIV and TB: update on current research efforts.. Current HIV Res.

[19] Pettifor A, MacPhail C, Corneli A, Sibeko J, Kamanga G (2011). Continued high risk sexual behavior following diagnosis with acute HIV infection in South Africa and Malawi: implications for prevention.. AIDS Behav.

[20] Robb M (2011). The Early Capture HIV Cohort Study (ECHO): prospective identification of acute HIV infection prior to peak viremia among high-risk populations [poster]..

[21] Fiebig EW, Wright DJ, Rawal BD, Garrett PE, Schumacher RT (2003). Dynamics of HIV viremia and antibody seroconversion in plasma donors: implications for diagnosis and staging of primary HIV infection.. AIDS.

[22] Attia S, Egger M, Muller M, Zwahlen M, Low N (2009). Sexual transmission of HIV according to viral load and antiretroviral therapy: systematic review and meta-analysis.. AIDS.

[23] Donnell D, Baeten JM, Kiarie J, Thomas KK, Stevens W (2010). Heterosexual HIV-1 transmission after initiation of antiretroviral therapy: a prospective cohort analysis.. Lancet.

[24] Lingappa JR, Hughes JP, Wang RS, Baeten JM, Celum C (2010). Estimating the impact of plasma HIV-1 RNA reductions on heterosexual HIV-1 transmission risk.. PLoS ONE.

[25] Williams B, Lima V, Gouws E (2011). Modelling the impact of antiretroviral therapy on the epidemic of HIV.. Curr HIV Res.

[26] Fraser C, Hollingsworth TD, Chapman R, de Wolf F, Hanage WP (2007). Variation in HIV-1 set-point viral load: epidemiological analysis and an evolutionary hypothesis.. Proc Natl Acad Sci U S A.

[27] Williams BG (2011). How important is the acute phase in HIV epidemiology? arXiv.. http://arxiv.org/pdf/1105.2767v1.pdf.

[28] Williams BG, Dransfield RD, Brightwell R (1990). Tsetse-fly (Diptera, Glossinidae) population-dynamics and the estimation of mortality-rates from life-table data.. Bull Entomol Res.

[29] Lotka AJ (1907). Relation between birth rates and death rates.. Science.

[30] Goosby E (2011). An inspiring vision: creating an AIDS-free generation..

[31] Clinton HR (2011 Nov 8). Remarks on ‘creating an AIDS-free generation’.

[32] Jacquez JA, Koopman JS, Simon CP, Longini IM (1994). Role of the primary infection in epidemics of HIV infection in gay cohorts.. J Acquire Immune Defic Syndr.

[33] Eaton JW, Hallett TB, Garnett GP (2011). Concurrent sexual partnerships and primary HIV infection: a critical interaction.. AIDS Behav.

[34] Koopman JS, Jacquez JA, Welch GW, Simon CP, Foxman B (1997). The role of early HIV infection in the spread of HIV through populations.. J Acquir Immune Defic Syndr Hum Retrovirol.

[35] Wallinga J, Lipsitch M (2007). How generation intervals shape the relationship between growth rates and reproductive numbers.. Proc Biol Sci.

[36] Grassly NC, Fraser C (2008). Mathematical models of infectious disease transmission.. Nat Rev Microbiol.

[37] Hallett TB, Singh K, Smith JA, White RG, Abu-Raddad LJ (2008). Understanding the impact of male circumcision interventions on the spread of HIV in southern Africa.. PLoS ONE.

[38] Boily MC, Mâsse B, Alsallaq R, Padian NS, Eaton JW (2012). HIV treatment as prevention: considerations in the design, conduct, and analysis of cluster randomized controlled trials of combination HIV prevention.. PLoS Med.

